# Gender equality in academic science

**DOI:** 10.1002/1873-3468.13056

**Published:** 2018-04-27

**Authors:** Laura Norton

**Affiliations:** ^1^ Babraham Institute Cambridge UK

A woman walks into a hotel restaurant

Server: Can I take your room number please?

Woman: Yes, 317

Server: Sorry, can I check that again please?

Woman: Yes, 317

Server: Are you sure? Oh, you must be staying with the professor.

I was told this story at a meeting on International Woman's Day 2018, it had occurred the evening before. Despite recent progress showing that children are now more often depicting women in drawings of scientists 1 it seems as adults our first unconscious thought is still that a professor is a man.

In Europe, approximately half of PhD graduates are women, in life sciences women account for greater than 50% 2. However, there is a dramatic drop in representation of women at leadership levels, with only 20% of grade A researchers and 15% of Directors of Institutions being women.

## Does this matter?

Despite the fact that gender equality is morally right and the fact that we are losing valuable talent and expertise, ultimately we need to carry out the best science. For this to happen we need a diversity of inputs and talents. If we are all the same, if we think in the same way, our science will be limited. The huge challenge of understanding the world around us demands input from different perspectives. If we recognize the need for broad input to ensure that our science is the best it can be, then we need to create an inclusive and motivating environment that science and each individual will benefit from.

## Why?

Why don't we see the same proportion of women at leadership level as at PhD level? Are women not capable? Do women not have a desire to progress? Or are there barriers preventing women from reaching senior levels?

If you are brave enough to voice your belief that the issue is with women's competence or drive, there is plenty of accumulating literature (too much to cite here; for a starting point see http://www.eige.europa.eu) that will show these are most likely not the answers. However, there is plenty of evidence to show that barriers and discrimination have impeded women's progression for too long.

## What are the barriers?

**Figure 1:**
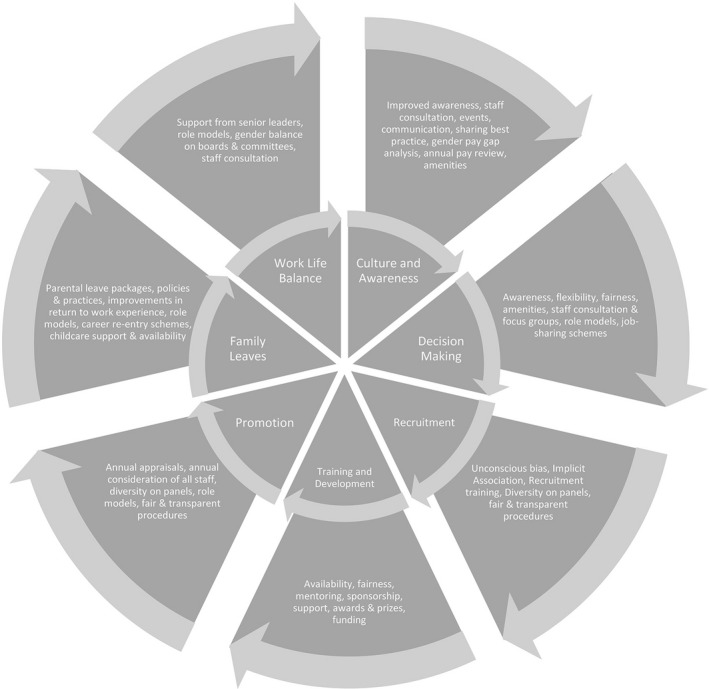
Barriers and actions to improve gender equality.

Barriers stem from the culture and stereotypes that most of us have unknowingly been subjected to through society since birth. In order to start pushing through these barriers we need to break them down into more manageable pieces. Areas important to address are: decision‐making, recruitment, training and development, promotion, family leaves, work‐life balance, culture and awareness. (Fig. 1).

Focusing on *recruitment* there are many areas where bias could be introduced. Who is making the decisions and what are their unconscious biases? Is the process fair, open and transparent? Is any language or images used in the advert, job description or institutions webpages that would lead to one group of people being favoured or believing they are more favoured than another? Many of these questions can be asked of other processes in academic science such as peer evaluation.

## What can we do?

### Collective responsibility is key to change

No one is to blame for what has passed but we all have responsibility to act now to make positive changes for the future. Both institutions and individuals have the ability to create change. Even if you do not feel that your organization is doing a lot in this area there are things that you can do to help gender equality move forward.

### Awareness is imperative

We can all be aware of our subconscious biases – there are plenty of online tests (e.g. Harvard Implicit Association Test) and trainings available making us aware of our own biases and stereotypes that we may hold. Being aware allows us to question our own (and sometimes other people's) thought processes. Have we judged a situation or person fairly, have we used a conscious decision‐making process?

### Action should be evidence‐based

Organizations need to take steps to self‐reflect. We have heard many anecdotes, like the one at the start of this article, but change should be evidence‐driven. Institutions need to gather data, explore their policies and practices and fully understand how these impact upon individuals and their core business. We too as individuals have a collective responsibility to provide this data and opinions to drive change.

### Role models drive change

We should all lead by example, whether this is changing a practice in the workplace or altering behaviour at home. We all look to our seniors, whether this is someone in the workplace modelling their workplace behaviour on that of a senior manager, or a child at home following examples set by their parents.

### Sharing the load

By sharing responsibilities at home we enable equal participation in the work place.

*Sharing best practice* and working together in this area is vital for academic institutions and society to progress. The Babraham Institute, Cambridge, UK, are fully committed to improving gender equality in academic science. We're keen to learn from others and to share our own experiences.

### About the author

**Laura Norton** obtained her PhD from the University of Cambridge studying at the Babraham Institute before undertaking postdoctoral positions at the Paterson Institute of Cancer Research and at the Babraham Institute. She is currently the Athena SWAN (Scientific Women's Academic Network) manager and LIBRA (Leading Innovative Measure to Reach Gender Balance in Research) GEP (Gender Equality Plan) team leader at The Babraham Institute. The Athena SWAN charter, established by the British Equality Challenge Unit in 2005, encourages and recognizes commitment to advancing the careers of women in STEM (science, technology, engineering and mathematics) in higher education and research. LIBRA, a European project awarded within the H2020 EU call for Promoting Gender Equality in Research and Innovation, aims to increase the participation of women in leadership positions in the life sciences.
